# Effects of a Short-time health literacy promotion program (HeLPP) on biochemical factors, self-care and quality of life among rural patients with type-2 diabetes: A field trial with Solomon four-group design

**DOI:** 10.34172/hpp.42787

**Published:** 2024-07-29

**Authors:** Farzaneh Golboni, Hakim Ahmadzadeh, Haidar Nadrian, Towhid Babazadeh, Sarisa Najafi, Parvaneh Ghahremaninasab, Kamyar Pirehbabi, Haleh Heizomi, Hassan Mahmoodi

**Affiliations:** ^1^Ministry of Health and Medical Education (MOHME), Tehran, Iran.; ^2^Department of Health Education and Promotion, Faculty of Health, Tabriz University of Medical Sciences, Tabriz, Iran; ^3^Social Determinants of Health Research Center, Tabriz University of Medical Sciences, Tabriz, Iran; ^4^Department of Public Health, Sarab University of Medical Sciences, Sarab, Iran; ^5^Department of Psychology, Islamic Azad University-Sanandaj Branch, Sanandaj, Iran; ^6^Department of Gerontology, Faculty of Health, Tabriz University of Medical Sciences, Tabriz, Iran; ^7^Student Research Committee, Tabriz University of Medical Sciences, Tabriz, Iran; ^8^Department of Public Health, Tabriz University of Medical Sciences, Sarab, Iran; ^9^Department of Public Health, Kurdistan University of Medical Sciences, Sanandaj, Iran

**Keywords:** Type 2 diabetes mellitus, Health literacy, Quality of life, Self-care

## Abstract

**Background:**

Current evidence suggests that health literacy (HL) impacts self-care behaviors and quality of life (QOL) in patients with type 2 diabetes mellitus (T2DM). This study aimed to evaluate the impact of a short-time health literacy promotion program (HeLPP) on self-care behaviors and QOL in rural patients with type 2 diabetes.

**Methods:**

Conducted from 2018 to 2019 in Chaldoran county, Iran, this randomized controlled trial followed the Solomon four-group design. Participants included 160 rural individuals with T2DM, who were divided into two intervention (A and C) and two control (B and D) groups. Pre-tests were conducted for intervention group A and control group B, with post-tests administered to all groups at three and six months. Interventions, consisting of five training sessions lasting 45 to 55 minutes, were planned and executed based on pre-test analyses. Primary outcomes were QOL and self-care behaviors, and secondary outcomes were glycated hemoglobin (HbA1c), HL and patients’ awareness of the disease.

**Results:**

Prior to the intervention, there was no significant difference in awareness, HL, self-care behaviors, HbA1c, and QOL between intervention group A and control group B (*P*>0.05). However, at three and six months after the educational program, intervention group A exhibited significantly increased average scores in awareness, HL, self-care behaviors, and QOL, along with reduced HbA1c levels (*P*<0.05) compared to control group B. No interaction was detected between the pre-test and the primary and secondary outcome scores after intervention.

**Conclusion:**

Implementing intervention programs like HeLPP focusing on enhancing practical HL and empowering T2DM patients seems to be promising in improving patients’ self-care behaviors and QOL, while reducing their HbA1c levels.

**Trial Registration:**

Identifier: IRCT20131116015422N7; https://irct.behdasht.gov.ir/trial/35569.

## Introduction

 Type 2 diabetes mellitus (T2DM) is a chronic disease characterized by chronic hyperglycemia and impaired metabolism of carbohydrates and proteins due to deficiency or lack of insulin.^1^ The global prevalence of diabetes is estimated to be 10.2%, and projections suggest an increase to 10.9% by 2030 and 13.3% by 2045, in Iran.^2^ In a current study among an Iranian population, the overall prevalence of T2DM was reported to be 13.8%.^3^ Patients with T2DM are at risk of long-term complications, including micro-vascular and macro-vascular diseases, such as retinopathy, neuropathy, cardiovascular disease, and diabetic foot.^4,5^ Furthermore, T2DM can have psychological effects such as anxiety and depression, which can significantly impact patients’ quality of life (QOL).^4^ Several risk factors, including modifiable factors like obesity, diet, and exercise, contribute to the development of diabetes.^6^ Efforts to address these factors through lifestyle changes can help prevent or manage T2DM.^7,8^

 Self-care plays a crucial role in preventing and managing T2DM and improving the QOL among affected individuals. Poor self-care behavior is a significant challenge faced by healthcare providers, particularly in developing countries.^9^ Improved self-care behavior has been associated with better blood sugar control, as indicated by glycated hemoglobin (HbA1c) levels.^10^ Health literacy (HL) is another critical factor that influences self-care compliance and diabetes outcomes.

 The World Health Organization (WHO) defines HL as an individual’s cognitive and social skills to access, understand, and use health information effectively for promoting and maintaining good health.^11^ Inadequate HL has been linked to poorer glycemic control and higher rates of complications among adults with T2DM.^12^ A meta-study has shown that promoting HL can effectively improve QOL.^13^ Previous studies have demonstrated that knowledge about diabetes and adherence to medication and dietary recommendations can be improved among individuals with low HL, through methods such as the teach-back method and the use of pictorial images.^14^ For example, a study by Babamir Satehi et al revealed that teach-back and multimedia teaching interventions were effective in enhancing self-care in patients with diabetic foot ulcers.^15^ The teach-back technique, a communication tool used to improve understanding of complex information, has been found to be effective across various settings and populations.^16^

 Considering the significance of HL and self-care behaviors in patients with T2DM, as well as the importance of maintaining their QOL and preventing disease complications, it is crucial to implement integrated training programs. Despite the importance of HL education, there is a lack of quantitative studies investigating its impact on health behaviors and QOL in patients with T2DM. Evidence also emphasize the lack of effective protocols for patients with diabetes. ^17^ In 2018, we developed a protocol for a health literacy promotion program (HeLPP) to promote self-care behaviors and QOL among rural type 2 diabetic patients. Due to the low literacy level in rural diabetic patients,^18^ and their possible lack of accessibility and availability to healthcare services, we supposed that the rural patients with T2DM might be prone to lower HL levels, compared to their urban counterparts. So, we chose to select the rural patients with T2DM, as study population. The protocol and the results of the behavioral and educational assessment on the pre-test data are now published elsewhere.^19^ We report on the effects of the HeLPP on the primary and secondary outcomes in the rural patients with type 2 diabetes in Chaldoran county, Iran.

## Materials and Methods

###  Study design

 This study was a randomized controlled trial based on the Solomon four-group design, which was conducted from 2018 to 2019. This design helps to minimize the impact of confounding variables and enables researchers to evaluate the effects of pre-testing on measured outcomes.^20^ The protocol of the study is previously published elsewhere.^19^

###  Setting and participants

 Applying simple random sampling, a total of 180 rural patients with T2DM, who were registered in Chaldoran healthcare centers, West-Azarbaijan province, were invited to participate in the study. Inclusion criteria included the individuals diagnosed with type 2 diabetes by a general physician and having health records in the Chaldoran healthcare centers. Exclusion criteria involved hospitalization, mental disorders (e.g., dementia, Alzheimer’s, mental retardation), severe movement restrictions (e.g., limb disabilities), and debilitating cardiovascular diseases. Finally, 160 patients included in the study. The researcher contacted the eligible participants through available addresses or phone numbers from their health records and invited them to take part in the study.

 First each participant signed a detailed informed consent form. Then prior to the pre-test, they were randomly assigned to four groups, namely A, B, C, and D, with 40 participants in each group (two intervention groups: A and C, and two control groups: B and D). Group A received both the intervention and pre-test/post-test, group B underwent pre-test/post-test without intervention, group C received the intervention and post-test, and group D only participated in the post-test without receiving the intervention.

###  Sample size

 The minimum sample size was determined based on a study conducted by Kheradmand et al,^21^ considering a 95% confidence level, 80% test power, two-tailed test, and using G*Power software. The study aimed for a sample size of 40 participants per group, taking into account a mean (SD) of 11.1 (3.43) in the first group, 13.5 (3.82) in the second group, an effect size of Cohen d = 0.66, and taking into account the possibility of attrition. As sampling was performed from many centers, design effect was considered in calculating the sample size.

###  Study procedure and intervention 

**Figure 1:**
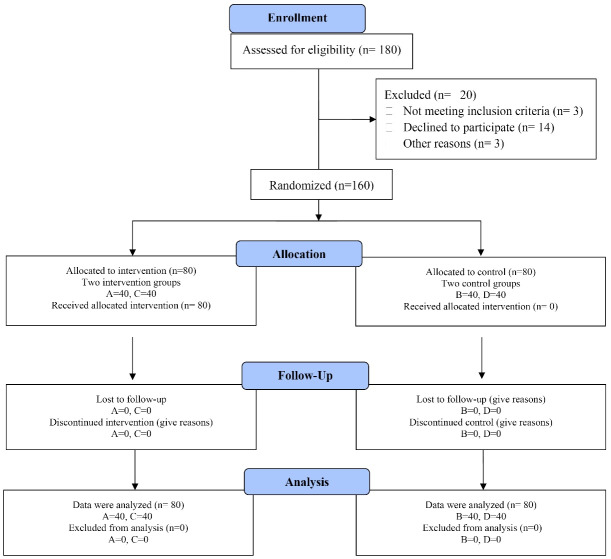
The procedure of the study from enrollment to data analysis. Group A received both the intervention and pre-test/post-test, group B underwent pre-test/post-test without intervention, group C received the intervention and post-test, and group D only participated in the post-test without receiving the intervention

 The HeLPP^19^ aimed to increase knowledge, promote HL, improve QOL, and enhance self-care behaviors. Strategies for increasing knowledge consisted of teaching strategies, group therapy, face-to-face teaching with the teach-back technique, and reminiscence. To promote HL, strategies included empowerment (improving knowledge of the disease and its control, enhancing reading skills, understanding and decision-making about the disease and self-care with the help of the teach-back technique). Strategies to improve self-care behavior involved behavior shaping, repetition, teach-back, and self-monitoring in self-help groups. Intervention strategies were formulated and implemented based on the data analysis of the pre-test,^19^ and based on the findings, the intervention activities were planned and executed during five training sessions of 45 to 55 minutes (see Table 1). Completing the HeLPP, we initiated the intervention. The study procedure is depicted in Figure 1. The details of the intervention protocol and the educational materials are published elsewhere.^19^ For more details of the materials, which were developed based on the CDC (Centers for Disease Control and Prevention) clear communication index,^22,23^ see Supplementary file 1. To comply with ethics, completing the implementation of the program, we prepared all participants in the intervention and control groups with the educational materials in a booklet format.

###  Data collection

 From February to September 2018, pre-test data were collected, through demographic questionnaires, a health literacy questionnaire, knowledge and self-care behavior questionnaires, diabetes QOL questionnaire, and the measurement of HbA1c and fasting blood sugar (FBS).

####  Demographic Data Questionnaire

 This questionnaire had 12 items on age, gender, ethnicity, marital status, education level, number of family members, income, duration of illness, history of other diseases, medication, smoking, and occupation.

####  Iranian Health Literacy Questionnaire (IHLQ)

 We applied the validated IHLQ developed by Haghdoost et al.^24^ This questionnaire has five dimensions: access (example: Can you obtain the health information you need from various sources?), reading skills (example: Is it easy for you to read educational materials about health?(, comprehension (example: Do you understand the explanations given by the doctor regarding your illness?(, evaluation (example: Can you evaluate the accuracy of health-related information available on the Internet?), and decision-making (example: Do you know where or whom to go to in the case you observe symptoms of a disease?).

####  Knowledge and Self-care Behaviors Scale

 A valid knowledge and self-care behaviors questionnaire developed by Didarloo et al^25^ was used in this study. The Cronbach’s alpha for this questionnaire was 0.83, indicating good internal consistency. The questionnaire consisted of two sections: an awareness questionnaire (What are the early complications of diabetes?), and a self-management behavior questionnaire. The self-management behavior questionnaire included four dimensions: diet (How many days in the last week did you follow the diet plan recommended by the doctors?), physical activity (How many days in the past week did you do the recommended amount of physical activity?), medication use (How many days did you take the recommended diabetes pills last week?), and blood sugar testing (How many days did you test your blood sugar last week?).

####  Diabetes Quality of Life Questionnaire

 A valid tool for assessing diabetes QOL, developed by Nasihatkon et al,^26^ was used in this study. The Cronbach’s alpha and intra-class correlation coefficient (ICC) of the scale in their study were 0.77 and 0.77, respectively. The scoring is based on a 5-point Likert-type scaling from 1= totally unsatisfied to 5 = totally satisfied. The questionnaire included 15 questions for both type 1 and type 2 diabetic patients. It assessed two dimensions: patient care behaviors (How often do you feel physically ill?) and satisfaction with disease control (How satisfied are you with the current status of your diabetes treatment?).

####  Biochemical assessments

 Five milliliters of fasting venous blood samples were collected from the participants in the Chaldoran Hospital laboratory. The blood samples were collected in gel tubes without anticoagulant and were then centrifuged for 10 minutes at a speed of 3000 rpm. HbA1c was measured using the HPLC method and a column chromatography kit with the laboratory glycosylated hemoglobin analyzer model BT3000 (made in Italy). The normal range of HbA1c for healthy individuals is less than 7.5%, and in people with diabetes, the treatment goal is to reduce and maintain levels below 7%. Serum levels of FBS were assessed using the spectrophotometry model Alcyon 300 and the Pars Azmon kit.

####  Anthropometric assessments

 Weight was measured using a digital scale (Seca 707, Haburg, Germany) with an accuracy of 100 grams. Measurements were taken while participants were wearing light clothing and not wearing shoes. Height was measured using a wall gauge (Seca, Haburg, Germany) without shoes, with an accuracy of 0.1 cm. Body mass index (BMI) was calculated by dividing weight (in kilograms) by the square of height (in meters). All measurements were conducted by a single person.

###  Primary and secondary outcomes 

 HbA1c and QOL were included in primary outcomes in the trial. Secondary outcomes were self-care behaviors, HL, awareness, and FBS.

###  Data analysis

 To describe the socio-demographic characteristics, HL, awareness, self-care behavior, QOL, and biochemical elements we used descriptive statistics, including frequency distribution, percentage, and measures of central tendency and dispersion (such as mean and standard deviation). The normal distribution of all variables was confirmed using the Kolmogorov-Smirnov test. The demographic characteristics of the participating patients were analyzed using the chi-square statistical test. The independent t-test was employed to determine the differences in mean scores of knowledge, HL, self-care behavior, QOL, HbA1c, and FBS in the baseline between the intervention and control groups. A repeated measurement t-test was used to measure the differences in mean scores within the groups before and after the training. Analysis of covariance (ANCOVA)was also applied to assess the impact of the intervention by adjusting baseline comparison and socio-demographic variables.

 All statistical analyses were performed using Statistical package for social sciences SPSS software (version 16, SPSS Inc., Chicago, IL, USA),^27^ and a *P* < 0.05 was considered statistically significant.

## Results

 The socio-demographic characteristics of the selected participants are presented in Table 2. The study included 160 people with diabetes who were divided into four groups (two intervention groups and two control groups). The majority of participants were in the age group of 61 years and older (56.3%), female (72.5%), of Turkish ethnicity (81.9%), non-smokers (83.8%), illiterate (68.8%), married (73.1%), with an income level of less than $237 (78.1%), and housewives (71.8%). Most participants had a duration of diabetes less than 10 years (63.12%) and had other diseases in addition to diabetes (65%). Statistical analyses revealed no significant differences between the control and intervention groups in terms of age, gender, smoking status, level of education, marital status, occupation, and presence of other diseases. However, we found a significant difference between the intervention and control groups in terms of ethnicity, income, and duration of diabetes (*P*<0.001) (Table 2).

 The independent t-test revealed no statistical difference between groups for HL, awareness, self-care behaviors, QOL, FBS, and HbA1c in the pretest (Table 3). However, the comparison of post-intervention scores between the four groups for all research variables (HL, awareness, self-care, QOL) showed significant differences, except for FBS (Table 4). Furthermore, the comparison of pre- and post-intervention mean scores (at 3 and 6 months after the intervention) using the repeated measurement indicated a significant difference in all variables in the intervention group.

 The implementation of HeLPP significantly increased the mean of HL, awareness, self-care behavior, and QOL in the intervention group at both 3 months and 6 months after the intervention, compared to before the intervention (*P*<0.001 in all). Additionally, the mean of HbA1c and FBS in the intervention group significantly decreased 3 (*P*<0.001) and 6 months (*P*<0.031) after the intervention, compared to before the intervention. In contrast, the comparison of pre- and post-scores between the control groups showed no significant statistical difference in all domains (Table 5). Moreover, examining the 3- and 6-month post-test scores for all primary and secondary variables, we found no significant trend of higher scores in the post-test only groups compared to the pre- and post-test groups (Table 5).

**Table 1:** Key characteristics of the health literacy promotion program (HeLPP)

**Title of sessions**	**Contents**	**Educational domain**	**Educational strategy**	**Time/team of educators**
Acquaintance with the program/Rising knowledge on the disease (40 min)	Acquaintance with the program	Cognitive	Lecture	5 min/health educationist (the first author)
	Type-2 diabetes and the role of blood sugar control	Cognitive	Lecture (5 min), self-help group discussion (2 min), feedback (2 min)	9 min/General physician of the health center
	Reasons for increasing/decreasing blood sugar	Cognitive	Lecture (5 min), self-help group discussion (2 min), feedback (2 min)	9 min/healthcare provider at the health center
	Signs/symptoms of low/high blood sugar	Cognitive	Lecture (2 min), self-help group discussion (2 min), story-telling (5 min)	9 min/General physician of the health center
	Conclusion	Cognitive	Teach-back (8 min)	8 min/both the general physician of the health center and the health educationist
Rising knowledge on the disease self-care and healthy lifestyle (45 min)	A brief review of the contents presented in the previous session	Cognitive	Lecture, questioning, and feedback (5 min)	5 min/health educationist (the first author)
	Healthy eating behaviors in type-2 diabetes/useful/harmful foods	Cognitive/ Attitudinal	Lecture (4 min), self-help group discussion (2 min), feedback (2 min)	8 min/nutrition care provider and the health educationist
	The role of regular medication taking and physical activity in the disease management	Cognitive/ Attitudinal	Lecture (5 min), self-help group discussion (2 min), feedback (2 min)	9 min/general physician and health educationist
	Disease complications	Cognitive/ Attitudinal	Lecture (5 min), self-help group discussion (2 min), feedback (2 min)	9 min/General physician of the health center and health care provider
	How to prevent disease complications in foot and eyes	Cognitive/ Attitudinal	Lecture (3 min), self-help group discussion (3 min), story-telling (3 min)	9 min/healthcare provider and the health educationist
	Conclusion	Cognitive/ Attitudinal	Teach-back (5 min)	5 min/the health educationist
Health literacy promotion (Reading skills and comprehension) (46 min)	A brief review of the contents presented in the previous session	Cognitive	Lecture, questioning, and feedback (4 min)	4 min/health educationist (the first author)
	Reading and interpreting a series of posters related to main self-care behaviors of type-2 diabetes	Cognitive/ Attitudinal/ Skill reinforcement	Lecture (1 min), self-help group discussion (4 min), feedback (3 min)	8 min/nutrition care provider, healthcare provider, and the health educationist
	Reading and describing the symbols, signs, and contents written on the signposts in hospitals and health centers	Cognitive/ Attitudinal/ Skill reinforcement	Lecture (1 min), self-help group discussion (4 min), feedback (3 min)	8 min/healthcare provider and the health educationist
	Descriptions on the possible recommendations of a doctor/the signs written on the medications by pharmacy	Cognitive/ Attitudinal/ Skill reinforcement	Lecture (3 min), self-help group discussion (4 min), feedback (2 min)	9 min/General physician of the health center and health care provider
	Descriptions on the possible recommendations before having a sugar test	Cognitive/ Attitudinal/ Skill reinforcement	Lecture (3 min), self-help group discussion (4 min), feedback (2 min)	9 min/healthcare provider and the health educationist
	A review of the content	Cognitive/ Attitudinal/ Skill reinforcement	Teach-back (4 min), story-telling (4 min)	8 min/the health educationist
Health literacy promotion (Decision- making) (45 min)	A brief review of the contents presented in the previous session	Cognitive	Lecture, questioning, and feedback (3 min)	3 min/health educationist (the first author)
	Acquaintance with the health centers and hospitals that the patients can refer when needed	Cognitive/ Attitudinal/ Skill reinforcement	Lecture (1 min), self-help group discussion (4 min), feedback (3 min)	8 min/healthcare provider and the health educationist
	When a patient can change the way of taking medications/the role of regular medication taking	Cognitive/ Attitudinal/ Skill reinforcement	Lecture (1 min), self-help group discussion (4 min), feedback (3 min)	8 min/General physician, healthcare provider, and the health educationist
	The criteria that one may pay attention to while buying dairy and foods (e.g. value of nutrients, fat, salt, and sugar)	Cognitive/ Attitudinal/ Skill reinforcement	Lecture (3 min), self-help group discussion (4 min), feedback (2 min)	9 min/General physician of the health center and health care provider
	The reasons for having regular checkups/how to decide when coming across to the behaviors that increase blood pressure	Cognitive/ Attitudinal/ Skill reinforcement	Lecture (3 min), self-help group discussion (4 min), feedback (2 min)	9 min/nutrition care provider, healthcare provider, and the health educationist
	A review of the content	Cognitive/ Attitudinal/ Skill reinforcement	Teach-back (4 min), story-telling (4 min)	8 min/the health educationist

**Table 2:** Differences in socio-demographic characteristics by the four groups, before the intervention

**Variable**		**Intervention group No. (%)**		**Control group No. (%)**		***P*** ** value**
		**A** ^*^	**C**	**B**	**D**	
		**n=40**	**n=40**	**n=40**	**n=40**	
Age (y)	<50	4(10.0)	12(30.0)	9(22.5)	7(17.5)	0. 300
	51-60	11(27.5)	11(27.5)	7(17.5)	9(22.5)	
	>60	25(62.5)	17(42.5)	24(60.0)	24(60.0)	
Gender	Male	14 (35.0)	10 (25.0)	12 (30.0)	8 (20.0)	0.474
	Female	26 (65.0)	30 (75.0)	28 (70.0)	32 (80.0)	
Ethnicity	Kurd	2 (5.0)	27 (67.5)	0 (0.00)	0 (0.00)	˂0.001
	Turk	38 (95.0)	13 (32.5)	40 (100)	40 (100.0)	
Smoking	Yes	8 (20.0)	11 (22.5)	3 (75.5)	4 (10.0)	0.057
	No	32 (80.0)	29 (72.5)	37 (92.5)	36 (90.0)	
Level of education	Illiterate	22 (55.0)	30 (75.0)	27(67.5)	31 (77.5)	0.127
	Elementary	18 (45.0)	10 (25.0)	13(32.5)	9 (22.5)	
Marital status	Married	24(60.0)	31(77.5)	30(75.0)	32(80.0)	0.219
	Single	2(5.0)	2(5.0)	0	0	
	Widow	14(35.0)	7(17.5)	10(25.0)	8(20.0)	
Income	<$237	40(100)	40(100)	27(67.5)	18(45.0)	˂0.001
	>$237	0	0	13(32.5)	22(55.0)	
Occupational status	Self-employed	0	0	1 (2.5)	0	0.641
	Employee	14 (35.5)	10 (25.0)	11 (27.5)	9 (22.5)	
	Housewife	26 (65.0)	30 (75.0)	28 (70.0)	31 (77.5)	
Suffering from other diseases	Yes	23 (57.5)	26 (65.0)	27 (67.5)	28 (70.0)	0.673
	No	17 (42.5)	14 (35.0)	13 (32.5)	12 (30.0)	
Duration of diabetes	<10 years	22 (55.0)	26 (65.0)	32 (80.0)	21 (52.5)	0.045
	10 years and more	18 (45.0)	14 (35.0)	8 (20.0)	19 (47.5)	

* Group A received both the intervention and pre-test/post-test, group B underwent pre-test/post-test without intervention, group C received the intervention and post-test, and group D only participated in the post-test without receiving the intervention.

**Table 3:** Mean differences in the primary and secondary outcomes before intervention, in groups A and B

**Variable**	**Groups**	**Mean**	**SD**	**MD**	**95% CI**	***P *** **value** ^a^
Health literacy	A	77.10	16.97	-3.075	-11.44 – 5.29	0.467
	B	80.17	20.45			
Awareness	A	16.35	3.42	0.425	0.063 – 1.21	0.607
	B	16.77	3.9			
Self-care behavior	A	24.64	6.59	0.61	-2.54 – 3.77	0.699
	B	24.02	7.47			
QOL	A	48.45	6.01	0.15	-2.86 – 2.56	0.913
	B	48.60	6.20			
FBS	A	135.31	29.51	6.63	-21.9 – 7.97	0.369
	B	141.94	34.57			
HbA-1C	A	7.52	1.14	0.28	-0.037 – 0.75	0.509
	B	7.33	1.33			

SD, Standard deviation; MD, Mean difference; CI, Confidence interval; FBS, fasting blood sugar; QOL, quality of life; HbA1c, glycated hemoglobin. Group A received both the intervention and pre-test/post-test, group B underwent pre-test/post-test without intervention. ^a^ Independent *t* test.

**Table 4:** Differences in the primary and secondary outcomes after intervention, between the four groups

**Variable**	**Groups**		***P *** **value** ^a^	***P *** **value** ^b^	***P *** **value** ^d^
Health literacy	A	C	0.976	0.005	1.00
		B	0.008		0.059
		D	0.005		0.044
	C	B	0.023		0.066
		D	0.017		0.049
Awareness	A	C	0.548	< 0.001^c^	0.930
		B	0.001		0.002
		D	0.001		<0.001
	C	B	0.001		0.002
		D	0.001		<0.001
Self-care	A	C	0.626	< 0.001	0.955
		B	0.005		0.031
		D	0.011		0.029
	C	B	0.001		0.007
		D	0.002		0.006
Quality of Life	A	C	0.719	0.001	0.985
		B	< 0.001		<0.001
		D	< 0.001		<0.001
	C	B	< 0.001		<0.001
		D	< 0.001		<0.001
FBS	A	C	0.379	0.322	
		B	0.082		
		D	0.082		
	C	B	0.511		
		D	0.671		
HbA-1C	A	C	0.171	0.005	0.658
		B	0.476		0.876
		D	0.020		0.082
	C	B	0.064		0.237
		D	< 0.001		0.003

Group A received both the intervention and pre-test/post-test, group B underwent pre-test/post-test without intervention, group C received the intervention and post-test, and group D only participated in the post-test without receiving the intervention. ^a^ Independent t-test; ^b^ ANOVA; ^c^ Welch; ^d^ Tukey.

**Table 5:** Mean (SD=standard deviation) differences in the outcome variables 3 and 6 months after intervention

**Variable**			**Pretest**		**3 months after the intervention**		**6 months after the intervention**		***P*** **value** ^b^
			**Mean**	**SD**	**Mean**	**SD**	**Mean**	**SD**	
Health Literacy	Intervention group	A^a^	77.10	16.97	91.67	14.00	91.50	13.91	˂0.001
		C			91.64	19.73	91.38	19.63	
		*P*		0.993		0.976		
	Control group	B	80.17	20.45	81.52	19.19	81.32	18.98	0.280
		D			81.05	18.88	80.85	18.80	
		*P*		0.911		0.912		
Awareness	Intervention group	A	16.35	3.42	21.40	2.3	21.20	2.35	˂0.001
		C			21.85	4.19	21.65	4.07	
		*P*		0.559		0.548		
	Control group	B	16.77	3.91	17.10	4.93	17.87	4.96	0.129
		D			16.10	3.58	16.35	3.04	
		*P*			0.303		0.102		
Self-care	Intervention group	A	24.64	6.59	34.33	8.48	30.33	9.92	˂0.001
		C			34.85	10.35	31.30	9.35	
		*P*			0.733		0.626		
	Control group	B	24.02	7.47	23.65	6.73	24.60	7.30	0.129
		D			24.07	9.01	24.55	9.70	
		*P*			0.812		0.979		
QOL	Intervention group	A	48.45	6.01	54.10	5.08	54.02	5.10	˂0.001
		C			55.05	7.70	54.52	7.12	
		*P*			0.517		0.719		
	Control group	B	48.60	6.20	47.85	6.15	48.12	5.53	0.408
		D			48.15	7.02	48.10	7.05	
		*P*			0.840		0.986		
HbA-1C	Intervention group	A	7.52	1.14	7.10	0.98	7.09	0.98	0.001
		C			6.82	0.87	6.83	0.83	
		*P*			0.183		0.171		
	Control group	B	7.33	331	7.31	1.28	7.30	1.28	0.093
		D			7.67	1.22	7.70	1.20	
		*P*			0.206		0.163		
FBS	Intervention group	A	135.31	29.51	129.05	30.18	129.28	30.07	0.031
		C			129.65	17.32	129.52	20.50	
		*P*			0.914		0.982		
	Control group	B	141.94	34.57	141.74	34.51	137.79	17.60	0.349
		D			139.25	21.05	138.95	21.03	
		*P*			0.699		0.799		

SD, Standard deviation; FBS, fasting blood sugar; QOL, quality of life; HbA1c, glycated hemoglobin. ^a^Group A received both the intervention and pre-test/post-test, group B underwent pre-test/post-test without intervention, group C received the intervention and post-test, and group D only participated in the post-test without receiving the intervention. ^b^ Repeated measures ANOVA.

## Discussion

 Our aim in this study was to investigate the effectiveness of a short-term HeLPP on QOL, HbA1c, FBS, HL, awareness, and self-care behaviors in rural patients with T2DM in Chaldoran County, Iran. After intervention, we found improvements in QOL, HbA1c, FBS, HL, awareness, and self-care behaviors of the patients.

 First of all, our findings highlighted the role of HeLLP in improving QOL in T2DM patients. A study on the effects of physical activity and healthy diet-based health education on the QOL of rural older people suggested that providing community-based health education interventions could be a promising public health strategy to improve QOL in old age.^28^ In addition, several interventional studies have highlighted the positive effects of educational programs on various aspects of QOL and overall well-being in patients with T2DM.^29,30^ Our findings are in line with those reported in other studies, which have indicated that diabetes education has a positive impact on improving the QOL score.^25,31^

 One important outcome of diabetes management is the improvement of HbA1c level. Our study showed that the HeLPP resulted in decreasing the levels of HbA1c in the patients. This is consistent with those reported by Kim and Hur who emphasized the relationship between self-care behaviors and metabolic control.^32^ Other studies have also shown a decrease in HbA1c levels following educational interventions.^32,33^

 The results of our study also showed effectiveness of the HeLPP on HL, which was similar to those found by Panahi et al, and Zhuang et al.^34,35^ As a health promotion program developed based on clear communication and teach-back strategies for rural patients with low literacy, the HeLPP showed to be promising in the promotion of HL and the improvement of awareness of health risks among these patients. In line with those found by Monami et al,^36^ increased awareness from the disease, its signs/symptoms, and complications is an important outcome of such interventions, considering that it serves as a precursor to behavior change. However, the effects of such interventions on patient’s awareness may vary depending on factors such as teaching methods and patient characteristics, as evidenced by Lowe and Bowen, who did not observe any increase in the awareness of diabetic patients after training.^37^ It is recommended that HL levels to be considered when designing educational interventions aiming at increasing patients’ awareness and self-care behaviors.

 In addition, the HeLPP had a significant impact on promoting self-care behaviors. Participants in the intervention group exhibited higher levels of self-care behaviors, compared to the control group, which was consistent with those found by Fransen et al.^38^ A previous systematic review found that nursing educational interventions improve glycemic control and self-care behaviors in patients with T2DM. Additionally, diabetes self-management plays a crucial role in improving glycemic control and reducing diabetes-related complications.^39^

 It is worthwhile to note that for all primary and secondary variables, we found no significant trend of higher scores in the post-test only groups compared to the pre- and post-test groups. This finding indicates that there was no potential negative effect of pre-test on the primary and secondary outcome scores after intervention in the present study. In health education interventions, it is commonly expected that pretest may interact with post-test score of intervention.^40,41^ Surveying individuals seems to persuade them for modifying their beliefs and behaviors, and may thus render them more resilient to such modifications.^42^

 It is also important to acknowledge some limitations of this study. The participants in our study were all selected from the patients inhabitant in rural areas of the county, which may affect the generalizability of the results Future studies should include larger sample sizes and more diverse populations to increase the generalizability of the findings. Due to time restrictions we had in finalizing the study, our follow-up was limited to 6 months. Therefore, longer follow-up periods are recommended to evaluate the long-term effectiveness of the program. It is also suggested that health centers and diabetes clinics prioritize coherent and planned education to improve HL and self-care behavior among patients with diabetes.

## Conclusion

 Overall, these findings demonstrate that implementing intervention programs like HeLPP focusing on enhancing practical HL and empowering rural patients with T2DM can be helpful in improving their awareness on the disease, HL, self-care behaviors, and QOL, while reducing HbA1c and FBS levels. Considering the specific characteristics of rural T2DM patients in developing countries (like low general literacy levels, and lack of accessibility and availability of healthcare services), we suggest that practitioners, community health nurses, and healthcare providers to integrate interactive and HL-associated strategies (like teach-back, self-help and clear communication strategies) into their approach when caring for the patients in such settings.

## Acknowledgments

 We thank the Faculty of Health Science, and all those who participated in this study.

## Competing Interests

 There is no conflict of interest in this study.

## Ethical Approval

 Before implementation the intervention each participant signed a detailed informed consent form. This study was approved by the Research Ethics Committee of Tabriz University of Medical Sciences (code: IR.TBZMED.REC. 1397.459).

## Supplementary Files


Supplementary file 1. Educational materials.
