# Supplementary material for: Effects of a Short-time health literacy promotion program (HeLPP) on biochemical factors, self-care and quality of life among rural patients with type-2 diabetes: A field trial with Solomon four-group design
Source: Health Promot Perspect. 2024 Jul 29;14(2):175–84. doi: 10.34172/hpp.42787 (PMC11403334; doi:10.34172/hpp.42787)
Supplement: Supplementary file 1 — Educational materials. [file hpp-14-175-s001.pdf]

Golboni et al, **Health Promotion Perspectives**, 2024, 14(2), 175-S1
doi: 10.34172/hpp.42787
https://hpp.tbzmed.ac.ir

Supplementary file 1: Educational materials

The educational materials were focused on the symptoms and complications of diabetes, control ways, how to prevent complications, physical activity, proper nutrition, when to do tests and take medicine, as well as how to properly perform self-care tasks prepared by them (Supplementary data file 1), which were prepared based on the clear communication strategies. These materials were prepared in a booklet format and delivered to all participants of intervention and control groups, after the implementation of the program.

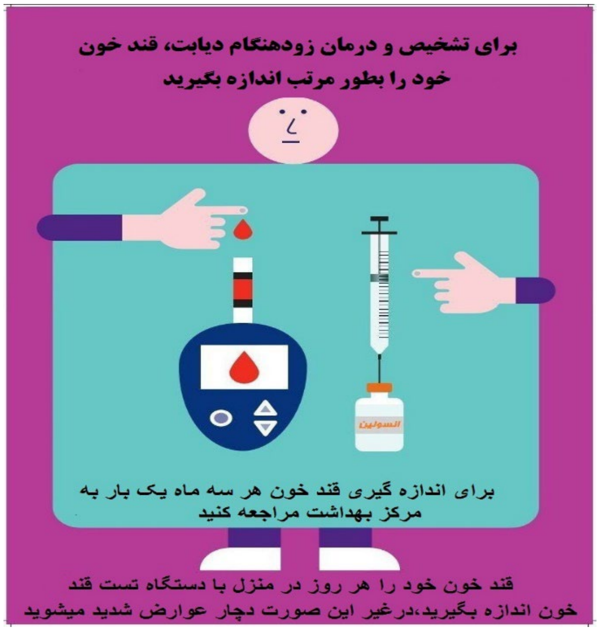

Poster no. 1: Poster of early diagnosis and treatment of diabetes. This poster shows the importance of measuring blood sugar as well as the times and places of the test in a visual way, and also points out the consequences and risks of not doing the test.

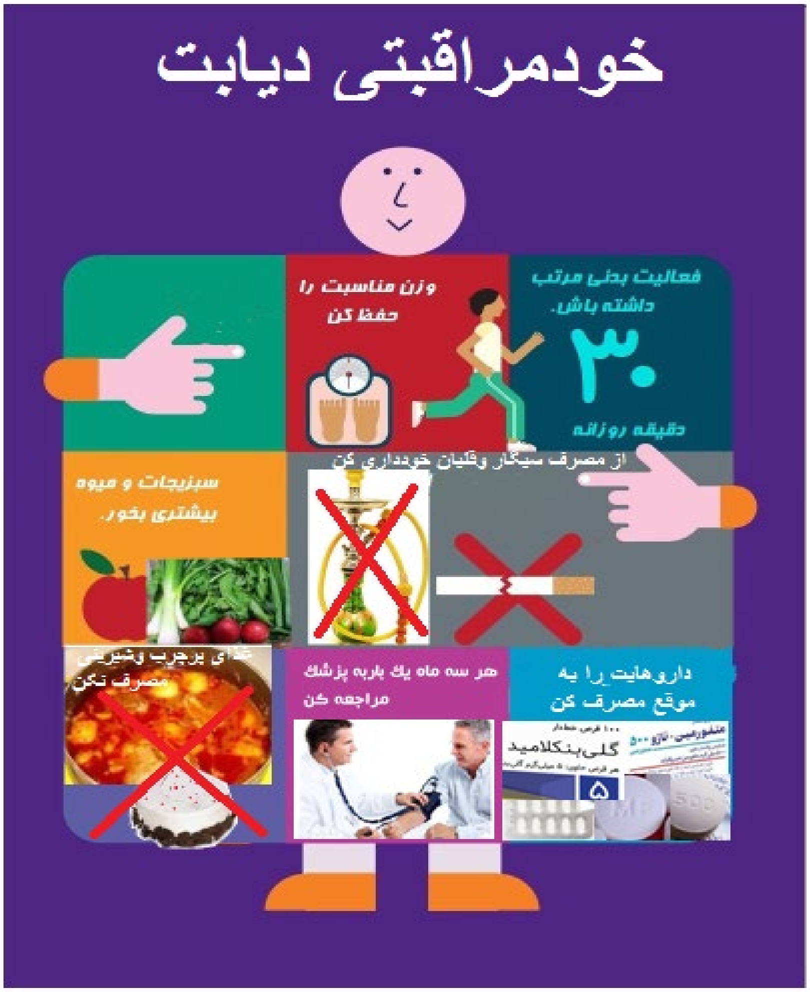

Poster no. 2. Diabetes self-care poster. This poster includes recommendations regarding physical activity, maintaining a proper weight, consuming fruits and vegetables, and not using tobacco and high-fat foods, and it also emphasizes regular use of medicines and tests.

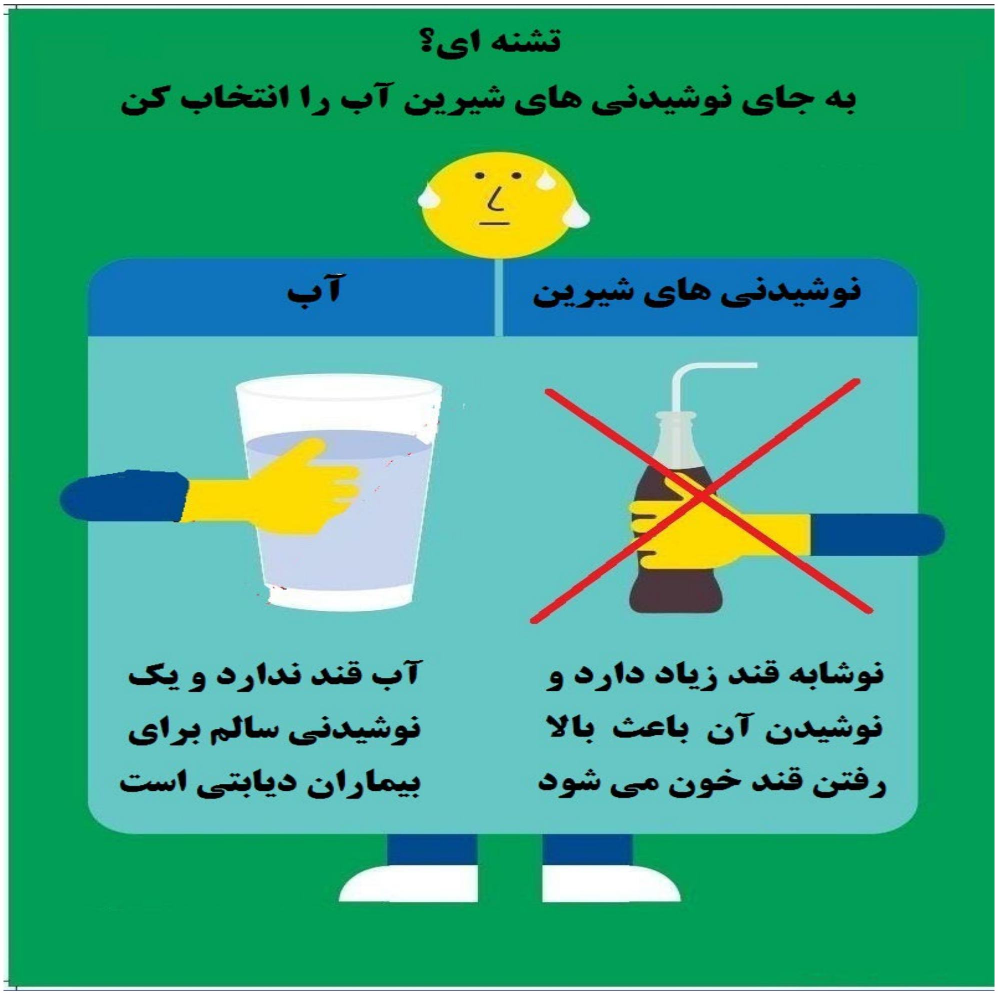

Poster no. 3. Poster of choosing the right drink. This poster advises in simple words and images that if a diabetic person is thirsty, water is the best option and emphasizes that other drinks, including soft drinks, are harmful for diabetic patients due to their sugar content.

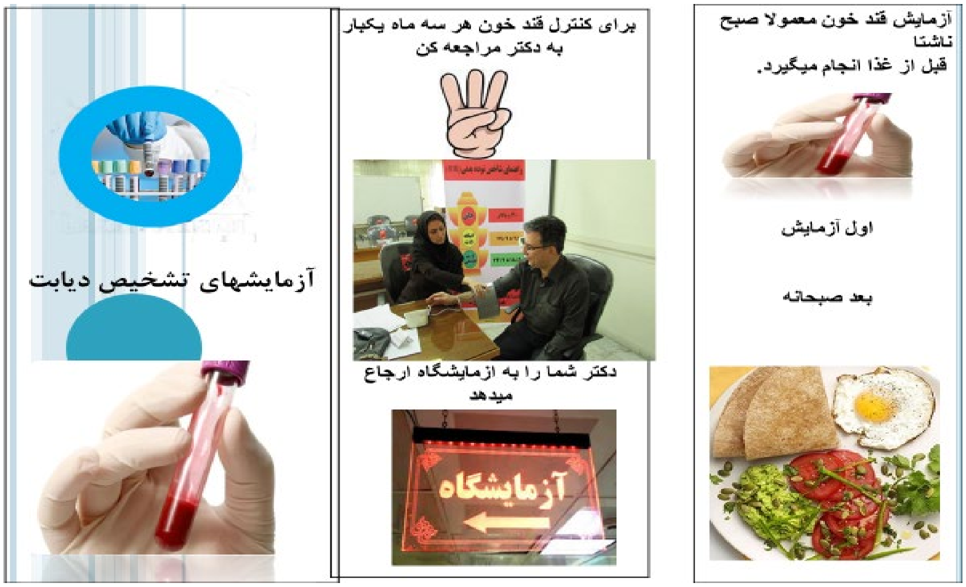

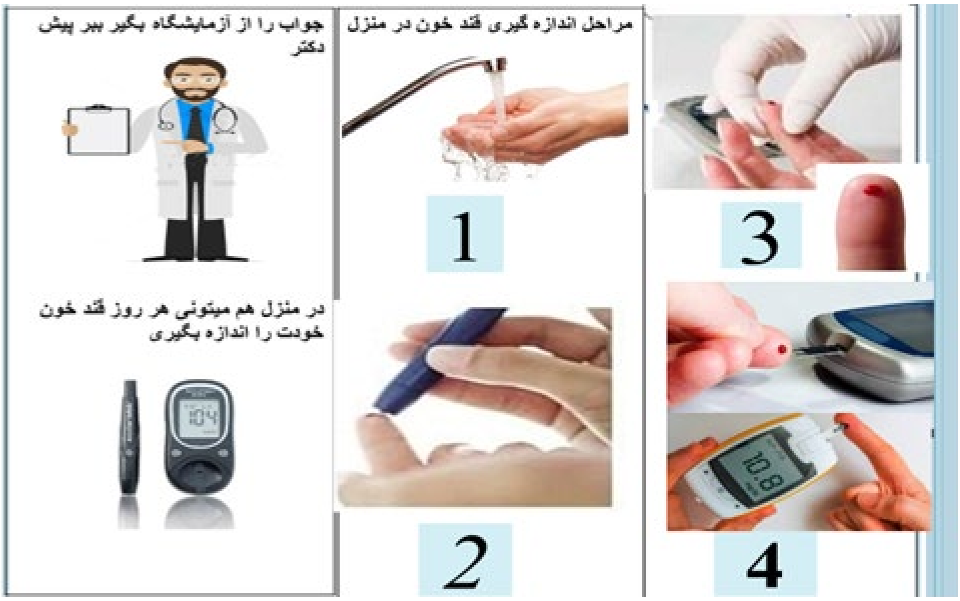

Pamphelet no. 1. Blood sugar test pamphlet. In this pictorial pamphlet, the diabetic patients were taught when to go for blood sugar test and it also specifies the place where the patient should go for the test. At the same time, it states that the patient should fast for the test. In the last pages, the stages of measuring blood sugar at home are presented in pictures.

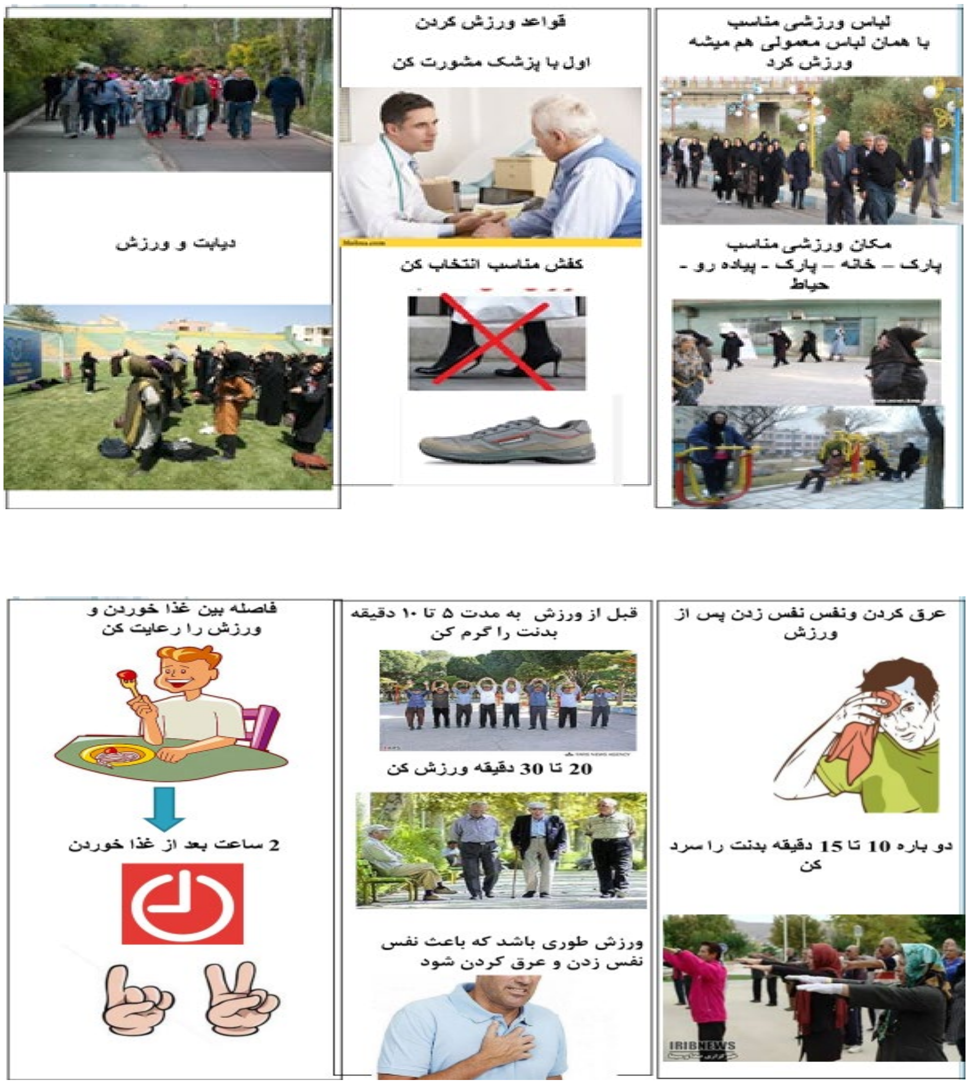

Pamphelet no. 2. Physical activity pamphlet. This material contains pictures of exercise and physical activity of the elderly and simple sentences that complement the pictures, and on the first pages, it states consulting a doctor before exercising, choosing clothes and shoes, and the appropriate place for exercise, then the appropriate distance between eating and It teaches exercise as well as the correct conditions and duration of exercise.

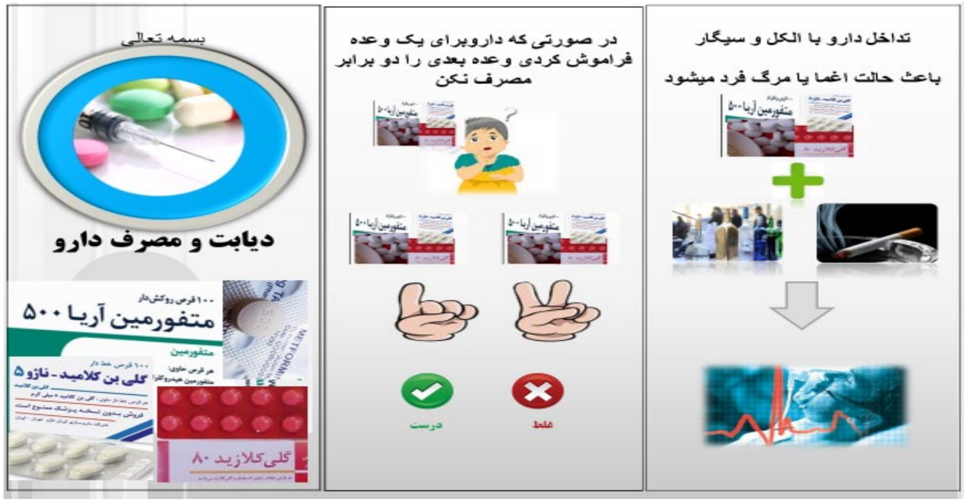

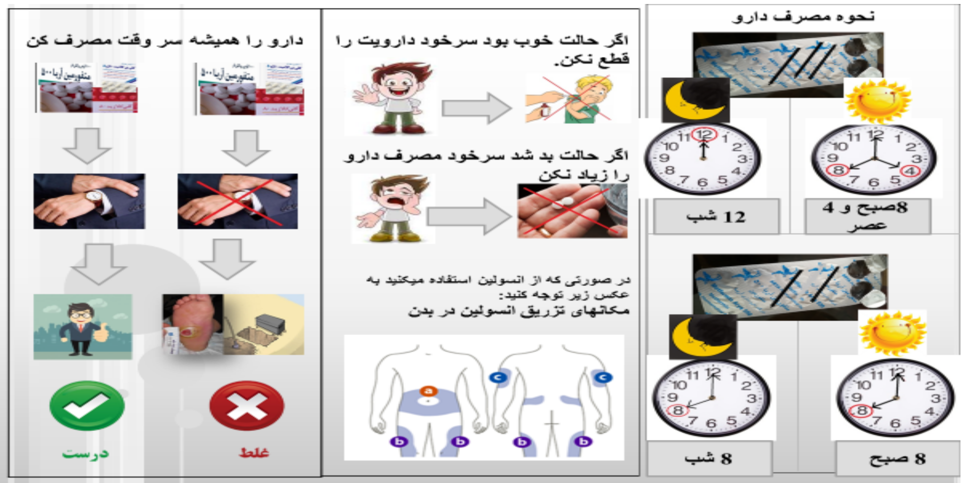

Pamphelet no. 3. Medication use pamphlet. In this pictorial pamphlet, the correct way to take the medicine is explained, and it is emphasized that the patient should not stop taking the medicine on his own, depending on whether the general condition is good or bad. Also, the way to take the medicine according to the doctor's order is illustrated in a picture for the patient's use and understanding.

1. Educational video. In this educational video, a movie of how a patient goes for a blood sugar test from the moment of entering the rural health center to the time of going to the diabetes unit are prepared, which are also shown in spoken form (in local language) at the same time as the movie is played, and the aim is to help the diabetic patient so that in case of referral from the village to the rural health center, the patient to be fully familiar with the process and procedures for conducting tests.
